# Pest Detection in Edible Crops at the Edge: An Implementation-Focused Review of Vision, Spectroscopy, and Sensors

**DOI:** 10.3390/s25216620

**Published:** 2025-10-28

**Authors:** Dennys Jhon Báez-Sánchez, Julio Montesdeoca, Brayan Saldarriaga-Mesa, Gaston Gaspoz, Santiago Tosetti, Flavio Capraro

**Affiliations:** 1 Universidad Politécnica Salesiana, Cuenca 010102, Ecuador; jmontesdeoca@ups.edu.ec; 2 Instituto de Automática (INAUT), UNSJ–CONICET, Av. Lib. Gral. San Martín 1109 (oeste), San Juan J5400ARL, Argentina; bsaldarriaga@inaut.unsj.edu.ar (B.S.-M.); ggaspoz@inaut.unsj.edu.ar (G.G.); stosetti@inaut.unsj.edu.ar (S.T.); fcapraro@inaut.unsj.edu.ar (F.C.)

**Keywords:** embedded sensing, edge AI, pest detection, edible crops, computer vision, hyperspectral spectroscopy, electronic nose, IoT, implementability, decision maps

## Abstract

**Highlights:**

**What are the main findings?**

- We introduced a modality-aware PCI rubric (performance–cost–implementability) with inter-rater κ to compare vision/AI, spectroscopy, and indirect sensor systems for pest detection in edible crops.
- We derived compact decision maps that translate PCI evidence into field-ready choices under the constraints of power, cost, maintenance, connectivity, and required action granularity.

**What is the implication of the main finding?**

- Practitioners can choose fit-for-purpose sensing modalities beyond accuracy-only benchmarks, improving the time to deployment.
- Reporting a minimum PCI metadata set enables reproducible, deployment-oriented comparisons across future studies.

**Abstract:**

Early pest detection in edible crops demands sensing solutions that can run at the edge under tight power, budget, and maintenance constraints. This review synthesizes peer-reviewed work (2015–2025) on three modality families—vision/AI, spectroscopy/imaging spectroscopy, and indirect sensors—restricted to edible crops and studies reporting some implementation or testing (*n* = 178; IEEE Xplore and Scopus). Each article was scored with a modality-aware performance–cost–implementability (PCI) rubric using category-specific weights, and the inter-reviewer reliability was quantified with weighted Cohen’s κ. We translated the evidence into compact decision maps for common deployment profiles (low-power rapid rollout; high-accuracy cost-flexible; and block-scale scouting). Across the corpus, vision/AI and well-engineered sensor systems more often reached deployment-leaning PCI (≥3.5: 32.0% and 33.3%, respectively) than spectroscopy (18.2%); the median PCI was 3.20 (AI), 3.17 (sensors), and 2.60 (spectroscopy). A Pareto analysis highlighted detector/attention models near (P,C,I)≈(4,5,4); sensor nodes spanning balanced (4,4,4) and ultra-lean (2,5,4) trade-offs; and the spectroscopy split between the early-warning strength (5,4,3) and portability (4,3,4). The inter-rater agreement was substantial for sensors and spectroscopy (pooled quadratic κ = 0.73–0.83; up to 0.93 by dimension) and modest for imaging/AI (PA vs. Author 2: $\kappa_{\mathrm{quadratic}}=0.30$–0.44), supporting rubric stability with adjacency-dominated disagreements. The decision maps operationalize these findings, helping practitioners select a fit-for-purpose modality and encouraging a minimum PCI metadata set to enable reproducible, deployment-oriented comparisons.

## 1. Introduction

Pest pressure remains a persistent drag on the yield, quality, and input efficiency in edible crops, making early detection and continuous monitoring essential pillars of precision agriculture and integrated pest management (IPM) [1,2]. Beyond farm-level losses, invasive and range-shifting species underscore the need for scalable, technology-enabled monitoring pipelines that can generalize across geographies and seasons [3]. Over roughly the last decade, three sensing umbrellas have matured for agricultural pest detection and monitoring: (i) vision-based systems using RGB imagery from fixed nodes, handhelds, or UAVs with classical CNNs, modern one-stage detectors (YOLO family), and transformer variants [4,5,6,7]; (ii) spectral and imaging spectroscopy (multispectral, hyperspectral, NIR/FTIR, fluorescence) that captures physio-biochemical cues preceding visible damage and extends to satellite/airborne remote sensing [8,9,10]; and (iii) indirect sensing (electronic noses/VOC sensing, acoustics/vibration, and IoT micro-stations) that infer pest activity via chemical or physical signatures [11,12,13].

Within the vision families, the work spans from curated benchmarks and lab conditions to increasingly realistic field deployments. Large-scale datasets such as IP102 catalyzed progress, but also made explicit the long-tail and domain-shift challenges typical of small targets in cluttered backgrounds [6]. Field-facing pipelines now include autonomous camera traps for insect counting and identification [7], embedded YOLO-style detectors tailored to constrained hardware in paddy fields [14], and architectures specialized for tiny pests (e.g., brown planthoppers) under dense foliage [15]. In row crops such as cotton, hybrid designs that combine efficient vision backbones with knowledge graphs illustrate a broader trend toward task-specific priors for decision support [16]. Parallel to vision, imaging spectroscopy has moved from controlled settings toward outdoor/operational use, e.g., the hyperspectral detection of early mite damage in cotton [9] and broader remote-sensing syntheses that situate pest signatures within vegetation indices and multi-sensor time series [8,10]. Indirect sensing modalities have also matured: e-nose platforms and VOC analytics offer compact, low-power options for enclosed environments or proximal sensing [12], bioacoustics leverage the flight tones and stridulation with modern deep learning [13], and IoT architectures integrate environmental context, communications, and fleet management [2,11].

Multiple umbrella reviews already cover these three modalities: comprehensive surveys of deep learning for crop pests [4,5], spectroscopy-centric overviews [8,10], and syntheses of sensor/IoT-based monitoring [11,12,13]. However, these are predominantly performance-centric: they benchmark models or instruments using in-distribution datasets and laboratory protocols, emphasizing accuracy, mAP, F1, and related metrics. In contrast, the determinants of adoption in farms, the on-device compute class and power envelope, the acquisition workflow and calibration stability, the enclosure/IP rating and environmental robustness, the communications and fleet operations, and seasonal maintenance are often under-specified or incomparable across studies [4,8,11]. The gap widens as pipelines incorporate heavier temporal models (e.g., hybrid transformer–ConvLSTM for pest forecasting) whose runtime and memory footprints complicate edge deployment, despite promising predictive value [17]. Practitioners thus face a practical question: not “what is the single best algorithm/instrument,” but “which sensing modality is realistically fit for purpose under budget, energy, labour, and connectivity constraints?”

This review addresses that gap from an embedded-engineering standpoint for edible crops. Instead of ranking algorithms or instruments in absolute terms, we organized the ecosystem by the sensing modality (vision/AI, spectroscopy, indirect sensing) and evaluated works through a modality-aware performance–cost–implementability (PCI) lens that explicitly elevates the implementability, the feasibility of running, maintaining, and scaling a solution in real environments. Concretely, implementability in our use comprises the following: (i) the inference compute class and power/thermal envelope (MCU/SoC/edge GPU); (ii) the acquisition workflow and calibration (illumination control, reflectance/white references, warm-up times); (iii) the enclosure and environmental robustness (dust, wind, condensation, vibration); (iv) communications/backhaul and fleet operations (LoRa/cellular/Wi-Fi duty-cycling, synchronization, over-the-air updates); and (v) seasonal maintenance overhead. These factors cut across modalities and directly condition the viability of on-farm deployments, from solar-powered camera nodes [7,14] to spectral rigs susceptible to illumination drift [8,9] and IoT traps requiring robust duty cycles and remote management [2,11].

Methodologically, we performed a systematic screening of peer-reviewed literature (2015–2025) restricted to edible crops and to studies with some degree of implementation/testing. Within each modality, we scored the selected works for the PCI and applied category-specific weights to reflect typical deployment trade-offs (e.g., higher emphasis on implementability for spectroscopy). To improve the transparency and reproducibility beyond accuracy-only reporting, we assessed the inter-reviewer agreement using weighted quadratic Cohen’s κ [18] on the PCI components and synthesized the evidence into compact decision maps that help choose a modality under common field constraint profiles (e.g., limited power with intermittent connectivity vs. high-throughput scouting with controlled illumination). In doing so, we aimed to complement performance-centered syntheses [4,5] with deployment metadata from concrete systems, including tiny-object pipelines [15] and hybrid knowledge-driven solutions [16].

**Aims.** To focus the review and make the guidance testable, we addressed four objectives: (i) map pest-detection technologies by sensing modality with deployment metadata [1,10,11]; (ii) compare studies with a modality-aware PCI rubric that foregrounds implementability and cost, spanning vision datasets and field systems [6,7,14]; (iii) assess the inter-reviewer reliability (weighted quadratic κ) for PCI scoring; and (iv) deliver decision maps that guide modality selection under typical field constraints in edible crops, accounting for IoT, e-nose, and bioacoustic options alongside imaging spectroscopy and embedded vision [8,11,12,13].

## 2. Materials and Methods

### 2.1. Review Objective and Scope

This review aimed to identify, classify, and comparatively evaluate technological approaches for pest detection in agriculture, with a particular focus on their potential deployment in embedded systems. The review emphasizes the technical feasibility of each approach in real-world, resource-constrained environments, rather than from purely theoretical or performance-centric perspectives.

The scope was restricted to three major technological domains that have shown either academic prominence or commercial viability in recent years:

1. Detection via imaging systems: Approaches using visual data (e.g., RGB, UAV-based imaging) for pest detection, typically processed via convolutional neural networks (CNNs) or other deep learning models.
2. Spectral imaging techniques: Techniques that include hyperspectral, multispectral, and infrared imaging modalities, capable of capturing physiological or biochemical plant indicators.
3. Sensor-based systems: Strategies involving gas sensors, volatile organic compound (VOC) detectors, or environmental sensors that capture indirect indicators of pest activity.

While many of these systems leverage artificial intelligence (AI) for classification and inference, this review categorizes technologies based on the sensing modality rather than the computational technique employed.

These categories were selected based on two key considerations: (i) their relevance in current scientific literature and (ii) their compatibility with embedded or semi-autonomous platforms. In this context, an embedded system is defined as a purpose-specific hardware and software configuration capable of operating independently or semi-independently in an agricultural environment. This review deliberately excludes solutions that merely involve data acquisition with centralized/cloud-only processing unless the design is explicitly intended for later embedded integration. An exception was made for imaging/AI, for which we included dataset-validated studies that offer a clear and commonly used deployment path to embedded inference (e.g., one-stage YOLO detectors with exportable weights), even in the absence of a reported hardware prototype.

Rather than focusing exclusively on conventional performance metrics such as the classification accuracy, this review proposes a comparative framework grounded in technical dimensions relevant to embedded system integration. These include, but are not limited to, aspects such as the implementation feasibility and operational constraints. A detailed evaluation framework is introduced in a subsequent section to systematically compare technological solutions across the selected categories.

### 2.2. Information Sources and Search Strategy

To ensure comprehensive coverage of technological developments in pest detection, a structured literature search was performed using two leading bibliographic databases: IEEE Xplore and Scopus. These platforms were selected due to their strong indexing in embedded systems, artificial intelligence, sensor technologies, and agricultural applications.

The search was restricted to peer-reviewed journal articles and conference proceedings, published between 2015 and 2025, and written in English. Only final, published versions were included; preprints, theses, and other forms of gray literature were excluded.

**Figure 1:**
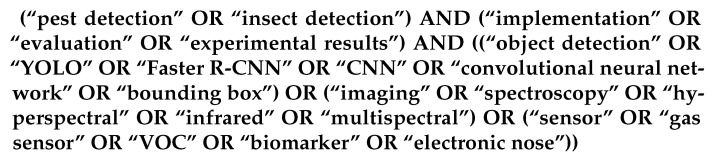
Main Boolean expression used to retrieve candidate articles.

A primary Boolean query was designed to retrieve works addressing pest or insect detection through image-based systems, spectral imaging, or sensor-based technologies. The query used was as follows (Figure 1):

The query shown in Figure 1 returned a total of 152 documents from IEEE Xplore and 240 from Scopus.

**Figure 2:**
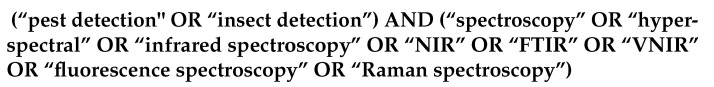
Supplementary Boolean expression focused on spectroscopy-related technologies.

To reinforce the coverage of spectral imaging technologies, an additional targeted search was conducted using the Boolean expression shown in Figure 2.

This supplementary search yielded 20 documents from IEEE Xplore and 36 from Scopus. Articles from the SPIE conference proceedings indexed in Scopus were excluded from the corpus due to restricted access limitations.

The complete set of results was exported and consolidated for further screening. A structured selection process inspired by the PRISMA framework was then applied to remove duplicates and assess article eligibility. Details on the screening strategy, inclusion/exclusion criteria, and review flow are provided in the next section.

### 2.3. Article Selection and Eligibility Criteria

The selection of articles followed a systematic process aligned with the PRISMA framework, focusing on the identification of studies that applied technological methods for pest or insect detection through computer vision, spectroscopy, or sensor-based systems.

The inclusion criteria were as follows:

- Peer-reviewed journal or conference articles published between 2015 and 2025.
- Written in English and available in final, full-text form.
- Focused on pest or insect detection in the context of agricultural applications.
- Employed one of the following approaches: (1) image-based systems (e.g., CNNs), (2) spectral imaging (e.g., hyperspectral or infrared), or (3) chemical/environmental sensors.

The exclusion criteria included the following:

- Studies dealing with plant diseases unrelated to pest activity.
- Articles focused on non-edible or industrial crops (e.g., cotton, wild plants).
- Duplicates across databases (IEEE and Scopus).
- Preliminary or inaccessible content (e.g., articles published only in SPIE, which were excluded due to access limitations).
- Studies lacking any form of implementation, testing, or system description, especially relevant for AI-based proposals.

All the exclusion criteria were systematically applied during both the title and abstract screening stages.

The primary search was conducted using the Boolean expression presented in Figure 1, which returned 152 results from IEEE Xplore and 240 from Scopus, totaling 392 records. After removing 20 duplicates from Scopus, 372 records were screened.

Title screening led to the exclusion of 138 articles, and abstract screening removed an additional 69, resulting in 165 articles included for the full analysis.

To reinforce the coverage of spectral imaging technologies, an additional targeted search was conducted using the Boolean expression shown in Figure 2. This returned 56 records (20 from IEEE Xplore and 36 from Scopus). After removing 13 duplicates, 43 records were screened. Of these, 3 could not be retrieved due to access limitations (SPIE publications), while 12 were excluded by title and 15 by abstract. The final contribution of this additional search was 13 spectroscopy-focused articles. To ensure a transparent and reproducible article selection process, the PRISMA framework was applied to both search strategies. The first query (Q1) was designed to broadly capture studies involving pest-detection technologies across computer vision, spectroscopy, and sensor-based systems. The second query (Q2) targeted spectral imaging techniques more specifically to reinforce coverage in that category.

**Figure 3:**
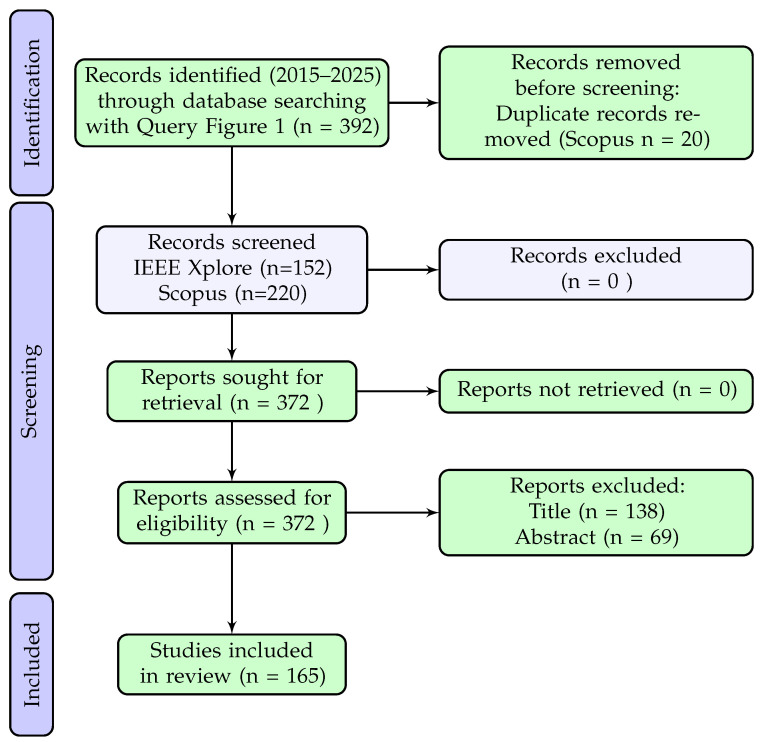
PRISMA statement [19].

**Figure 4:**
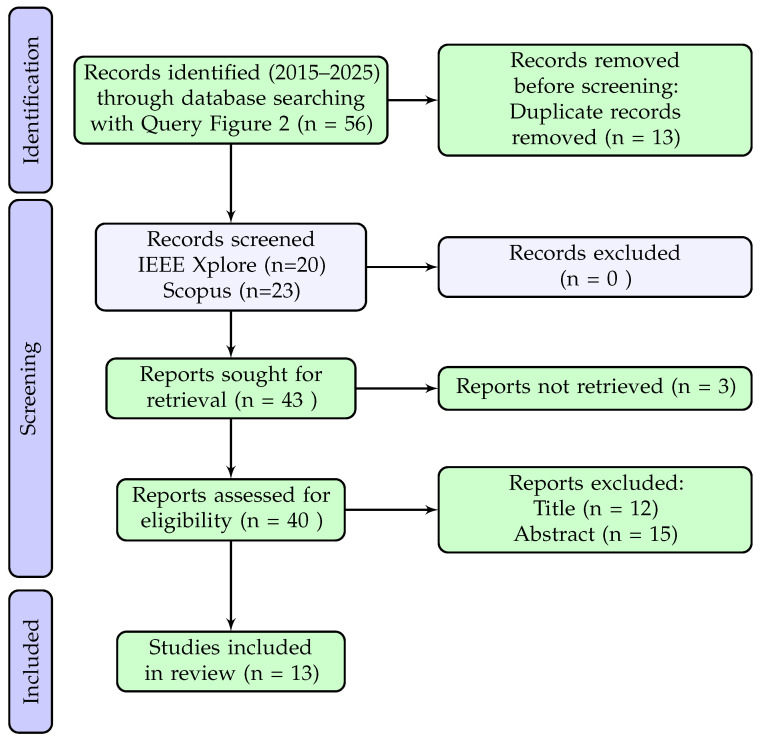
Additional PRISMA statement [19].

The following two PRISMA diagrams (Figure 3 and Figure 4) summarize the identification, screening, and inclusion stages for each query. The same inclusion and exclusion criteria were applied consistently to both searches, and the outcomes are shown independently to highlight the contribution of each query to the final article pool.

As shown in Figure 3 and Figure 4, the primary query (Q1) contributed a total of 165 unique articles, while the additional spectral-focused query (Q2) contributed 13 more. In both processes, duplicate records were removed prior to screening, and the same eligibility criteria were applied at the title and abstract levels. These criteria included the exclusion of studies on plant diseases unrelated to pest activity, non-edible or industrial crops, and non-implementable or inaccessible approaches.

Together, these searches yielded a combined total of 178 articles for technical evaluation. This merged dataset provided the foundation for the subsequent scoring and categorization by technological approach.

#### 2.3.1. Screening Procedure

The screening process was conducted in two stages (title and abstract), applying the same inclusion and exclusion criteria. Two independent reviewers participated in this process, both of whom are co-authors of this study. Disagreements regarding eligibility were discussed and resolved by consensus, ensuring the consistent application of the criteria across all records.

This dual-review strategy was employed to enhance the reliability and objectivity of the selection process, particularly in borderline cases where the article relevance was not immediately clear from the titles or abstracts.

#### 2.3.2. Inter-Rater Agreement (Weighted Cohen’s κ)

To quantify inter-reviewer reliability during screening and subsequent ordinal assessments, we used weighted Cohen’s κ on five-level scales (1–5). In Cohen’s formulation, the weighted version assigns smaller penalties to near disagreements and yields a chance-corrected proportion of a weighted agreement [18]. We report both the linearly and quadratically weighted κ, considering the quadratic version as the primary estimator for ordered categories.

Two independent comparisons were run to reflect complementary expertise: (i) the Primary Author (PA) vs. Author 1 in spectroscopy and sensors and (ii) the PA vs. Author 2 in imaging (AI). An agreement was computed per domain and per rating dimension.

Items within each domain were matched primarily by article title + domain. The five-category scale [1,…,5] was retained even if some categories were unused in a subset.

Let *O* be the observed agreement matrix (normalized to proportions) and *E* the chance-expected matrix from the product of the marginals. For a *k*-level ordinal scale (k=5 here) with weights $w_{ij}$ on cell (i,j), the estimator is

$$\kappa_{w}\;=\;1\;-\;\frac{\sum_{i,j}w_{ij}\;O_{ij}}{\sum_{i,j}w_{ij}\;E_{ij}},$$

with linear and quadratic disagreement weights

$$w_{ij}^{\mathrm{lin}}=\frac{\left|i-j\right|}{k-1},\;w_{ij}^{\mathrm{quad}}=\frac{{\left(i-j\right)}^{2}}{{\left(k-1\right)}^{2}}.$$

Following common practice for ordered categories, we treat quadratically weighted κ as the primary reliability index and linearly weighted κ as a sensitivity analysis [18].

A representative divergence occurred in the imaging (AI) category regarding the Pest-PVT study by Chen et al. [20], specifically on the cost axis. The Primary Author (PA) assigned a high score, reasoning that the architecture’s compact profile (24.74M parameters, 7.82 GFLOPs) suggested efficient inference compatible with a range of embedded platforms, even in the absence of explicit FPS metrics. The PA interpreted the reported Jetson TX2 deployment not as a strict minimum requirement, but as a representative embedded-class target, and inferred practical feasibility from the model’s reported complexity. In contrast, Author 2 applied a stricter reading, treating the TX2 as the baseline hardware assumption and discounting the score due to its relatively high cost in low-resource settings. The disagreement reflects distinct, but valid, interpretations of deployment framing: one emphasizing architectural efficiency and inferred generalizability, and the other prioritizing concrete evidence and direct affordability.

### 2.4. Article Categorization Framework

Given the diversity of technological approaches identified during the screening phase, the selected articles were first organized into three primary thematic categories based on their sensing modality and core technological principle:

1. Imaging-based systems: Studies focused on the application of algorithm-based machine learning or deep learning algorithms to visual data (e.g., RGB images, UAV footage) for pest detection.
2. Spectroscopy-based techniques: Articles employing spectral imaging methods such as near-infrared (NIR), hyperspectral, FTIR, or Raman spectroscopy to infer pest presence via biochemical or physiological indicators.
3. Sensor-based systems: Works describing the use of physical, chemical, or environmental sensors (e.g., gas sensors, VOC detectors, electronic noses) to capture indirect or non-visual indicators of pest activity. A few AI-heavy studies with a strong embedded component, where the primary focus is hardware/software integration, also fall under this category.

This thematic division was guided by both conceptual and practical considerations. Conceptually, these three categories represent distinct mechanisms of sensing. From an implementation perspective, they also differ significantly in terms of hardware integration, data acquisition workflows, and real-time processing feasibility, factors that are especially relevant in the context of embedded system deployment.

To ensure consistent classification, each article was analyzed based on its methodological description, focusing on the primary technology implemented rather than on keywords or title references. Articles with overlapping elements were assigned to the category most representative of their core sensing approach.

#### 2.4.1. Subcategorization Within Imaging-Based Studies

Due to the large number of articles in this group and their architectural diversity, a dedicated subcategorization scheme was applied to further refine the analysis. This technical classification grouped imaging-based studies into five subcategories based on the neural network architecture or computational model reported:

- CNN-based: Traditional convolutional neural networks used for image classification or object detection (e.g., AlexNet, VGG, ResNet).
- Hybrid CNN: Architectures combining CNNs with other mechanisms such as attention modules, recurrent units, or transformer-based components.
- YOLO-based: Studies implementing the you only look once (YOLO) family of models, often associated with practical deployments using PyTorch (.pt) formats.
- Transformer-based: Models employing Vision Transformer (ViT), Swin Transformer, or other self-attention-based frameworks for visual recognition tasks.
- Other methods: Approaches that did not fit any of the above categories, including non-standard or hybrid AI techniques.

This sub-classification enabled a more targeted evaluation of the architectural trade-offs, such as the computational requirements, training complexity, and deployment feasibility in constrained environments. It also allowed for a better comparison among solutions that share similar underlying principles, thereby enhancing the robustness of the analysis. To keep the comparative analysis tractable and technically consistent, within each AI subcategory, we restricted the comparison to the five most-cited papers (based on the citation count available at the time of retrieval). This decision responds to the size of the corpus, the strongly contextual nature of the performance criterion in PCI, and the need to preserve consistency and traceability in the scoring. The citation counts offer a reproducible proxy for influence, but may favour older or highly visible venues; this potential bias is acknowledged. All papers in the selected subset were evaluated using the same PCI rubric; studies outside this subset were not analyzed, and their omission should not be construed as a negative quality judgment.

To assess the practical relevance of the reviewed studies for deployment in embedded agricultural systems, a structured evaluation model was developed based on three key dimensions: performance (P), cost (C), and implementability (I). These three axes form the PCI framework, designed to go beyond accuracy-focused comparisons and capture the technological maturity and field-readiness of each system.

- Performance (P) refers to the system’s reported effectiveness in detecting pests or pest-related indicators. It encompasses the accuracy, sensitivity, or qualitative success as described by the authors.
- Cost (C) captures the economic burden associated with implementing the technology, based on hardware, instrumentation, and computational resources.
- Implementability (I) evaluates the feasibility of deploying the system in realistic agricultural scenarios, considering the robustness, portability, and compatibility with embedded or portable platforms.

Although PCI offers a unified evaluation structure, its application was adapted to reflect the specific nature of each technological domain, as detailed below. In all cases, implementability refers to the feasibility of deployment under real conditions; in imaging-based studies, this is proxied by factors such as real/semi-real validation and lightweight architectures, while in sensor and spectroscopy systems, it emphasizes robustness, calibration stability, and environmental tolerance.

#### 2.4.2. Adaptation of PCI to Imaging (AI)-Based Studies

Imaging (AI)-based approaches, primarily involving deep learning models, differ significantly from hardware systems in that their cost and complexity are largely tied to training and architecture design rather than physical implementation. The PCI dimensions were therefore interpreted as follows:

- Performance (P) was assessed using quantitative metrics reported by the authors, such as the classification accuracy, mean average precision (mAP), F1-score, recall, or precision. Object detection models were generally rated higher than classification-only models due to their greater operational relevance.
- Cost (C) focused on the computational requirements for inference. This included the type of hardware needed to deploy the model (e.g., microcontrollers, mobile devices, GPUs, TPUs). Training costs were excluded from consideration.
- Implementability (I) evaluated the feasibility of deployment under agricultural conditions, proxied in imaging studies by validation under realistic or semi-realistic settings (e.g., variable lighting, UAV imagery, real crop environments) rather than curated laboratory datasets. Lightweight architectures and field testing contributed positively to this score.

To reflect these priorities, the PCI dimensions for imaging (AI)-based studies were weighted as follows:

Performance: 0.60, Cost: 0.15, Implementability: 0.25

The chosen weights reflect how imaging-based studies are typically reported: performance dominates the available evidence, whereas implementability is often only partially demonstrated (e.g., proof of concept in field-like settings).

#### 2.4.3. Adaptation of PCI to Sensor- and Spectroscopy-Based Studies

For studies based on physical sensors and spectroscopic devices, the PCI model was applied with the following interpretations:

- Performance (P) evaluated the reported detection capabilities of the system, based on quantitative metrics (e.g., limit of detection, sensitivity, specificity) or qualitative success as described in trials. Whether the data were synthetic or collected in-field, the reported values were accepted at face value.
- Cost (C) accounted for the estimated economic cost of the required hardware, including sensors, spectrometers, or other measurement instruments. Systems with complex, laboratory-dependent instrumentation were rated lower in this dimension.
- Implementability (I) assessed the environmental and practical constraints involved in deploying the system. For example, a device requiring strict thermal stability or long warm-up periods was rated lower than a robust, low-maintenance system suitable for open-field conditions.

To reflect the differences between sensors and spectroscopic systems in terms of their complexity and inherent cost, distinct weighting schemes were applied:

- Sensor-based systems: P: 0.33, C: 0.33, I: 0.33.
  (Uniform weighting; used as baseline due to balanced technical and practical nature.)
- Spectroscopy-based systems: P: 0.40, C: 0.20, I: 0.40.
  (Cost was de-emphasized due to the intrinsically higher expense of these techniques; performance and feasibility were prioritized.)

These weighting choices reflect the different evidence profiles of the two domains. Sensor-based systems were assigned uniform weights (0.33/0.33/0.33) because the published works have typically balanced raw detection performance, component cost, and deployment feasibility in a similar proportion. In contrast, spectroscopy studies often report strong detection capabilities, but face recurring challenges in calibration stability, portability, and environmental robustness; therefore, implementability (0.40) was given equal importance to performance (0.40), while cost (0.20) was de-emphasized to account for the intrinsically higher baseline expense of spectroscopic instruments.

#### 2.4.4. Scoring and Normalization Strategy

All articles were scored on each PCI dimension using a discrete scale from 1 (lowest) to 5 (highest), resulting in an unweighted total score ranging from 3 to 15. These raw values reflect the internal merit of each system within its respective technological category.

To facilitate a comparison across domains with differing technical foundations, each score was subsequently normalized using the weighting schemes described above. This produced a weighted PCI score on a 1-to-5 scale, emphasizing deployment-oriented priorities specific to each category.

Formally, for each article, the final weighted score is given by the following:

(1) $$PCI_{\mathrm{final}}=w_{P}\;\cdot \;P+w_{C}\;\cdot \;C+w_{I}\;\cdot \;I$$

where $w_{P},w_{C},w_{I}$ denote the category-specific weights (see Section 2.4.1, Section 2.4.2 and Section 2.4.3), which sum to 1. The resulting normalized PCI scores form the basis for the comparative evaluation in Section 3, enabling a balanced assessment across heterogeneous technologies while preserving their intrinsic differences.

## 3. Results

### 3.1. PCI Scoring

In Table 1, Table 2 and Table 3, we report the raw PCI component scores (P,C,I∈{1,…,5}) assigned to each work, along with the resulting $PCI_{\mathrm{final}}$ calculated using the category-specific weights defined in Section 2.4.1—imaging (AI): 0.60/0.15/0.25; sensors: 1/3 each; and spectroscopy: 0.40/0.20/0.40. Thus, the tables present both the Primary Author’s (PA) base PCI ratings (P,C,I) and the corresponding weighted and normalized $PCI_{\mathrm{final}}$ score. The inter-rater reliability relative to co-raters is quantified in Section 2.3.2.

### 3.2. Aggregate Comparative Signals Across Sensing Domains

**Figure 5:**
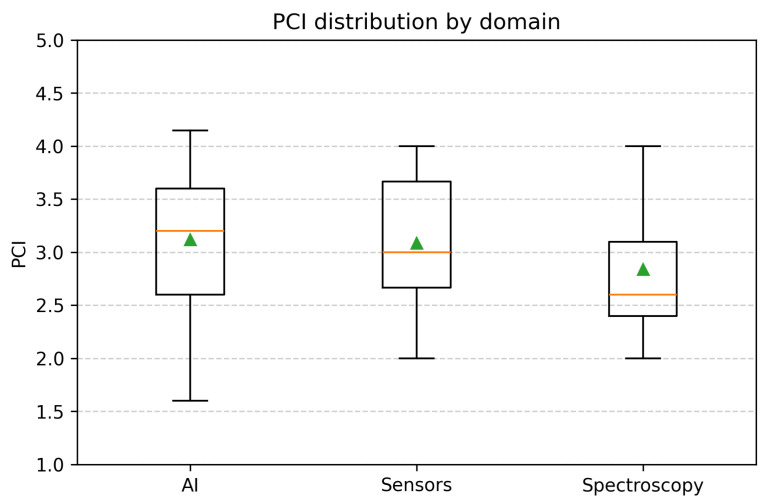
PCI distributions by domain (boxplots showing medians and means). The orange line indicates the median, and the green triangle indicates the mean. The y-axis is constrained to the rubric scale [1,5].

To complement the per-paper PCI tables, Figure 5 shows the full distribution of PCI scores by domain using boxplots (median, IQR, whiskers, and means).

#### 3.2.1. Imaging (AI) Subcategories

The ranking by median PCI presented in Table 4 shows that, when using CNN as a reference baseline, the median uplift is +1.00 for transformers, +0.80 for YOLO, +0.60 for H-CNN, and 0.00 for others. The dispersion is smallest for CNN (IQR =0.15) and largest for H-CNN (IQR =0.75), with YOLO and transformers showing intermediate variability (IQR =0.55 and 0.40, respectively). Ties are ordered by IQR and then by mean.

This ordering is consistent with the practical advantage of modern detectors and attention mechanisms for in-field inference and small-object sensitivity under variable conditions, which our implementability component emphasizes.

#### 3.2.2. Pareto Front Across Domains

Considering the 3D space (P,C,I), the Pareto sets (non-dominated) are compact: in imaging (AI), the two P=4,C=5,I=4 works remain; in sensors, (4,4,4) and (2,5,4) are mutually non-dominant (different trade-offs in capital cost versus performance); and in spectroscopy, (5,4,3) and (4,3,4) co-exist on the front, reflecting distinct balances between the raw detection capability and field portability. Under the PCI lens, deployable options cluster around modern AI detectors and purpose-built sensor traps, while spectroscopic pipelines, although promising for early or hard-to-visualize infestations, face integration frictions that lower their implementability scores. These results motivate the decision maps in the next section and the roadmaps that follow.

As shown in Table 5, the last column reports the proportion of studies with PCI≥3.5, a reference value introduced here as a preliminary indicator of deployability and further discussed in the following section.

### 3.3. Inter-Rater Agreement (PA vs. Author 1)

Weighted Cohen’s κ (linear and quadratic) by the domain and rating dimension is summarized in Table 6. Computation follows Section 2.3.2.

### 3.4. Inter-Rater Agreement (PA vs. Author 2)

Weighted Cohen’s κ (linear and quadratic) by the domain and rating dimension is summarized in Table 7. Computation follows Section 2.3.2.

## 4. Discussion

### 4.1. PCI Synthesis and Comparative Discussion

#### 4.1.1. At-a-Glance Signals Across Domains

The PCI tables and domain summaries in Figure 5 and Table 5 indicate a consistent ordering under the stated weights. Imaging (AI) and well-engineered sensor systems more frequently occupy the upper tail, while spectroscopy concentrates at mid-range values due to the implementability component.

#### 4.1.2. Imaging (AI) (Table 1)

Within AI, the median PCI increases when moving from classical CNNs to modern one-stage detectors and attention-based variants, as summarized in Table 4. Transformers showed the highest median (3.60) with low dispersion, reflecting their status as more complex and versatile architectures that consistently achieved strong scores across the board. YOLO models also achieved a relatively high median (3.40) with moderate variability, which is consistent with their role as a widely adopted “ready-to-use” solution that facilitates deployment and reproducibility. Hybrid CNNs present the largest IQR, indicating heterogeneous design choices and evidence settings. Lower PCI values in classical CNNs and “other” methods are linked to dataset-bound validations and less-generalizable evaluations.

#### 4.1.3. Sensor Systems (Table 2)

Two non-dominant lines achieved a competitive PCI. Balanced traps or stations with (4,4,4) co-existed with resource-constrained nodes (2,5,4) that traded raw performance for a favourable cost and implementability. Higher-scoring entries specify optics, enclosures, communications, duty-cycling, and fleet operations. Lower scores usually reflect limited operational validation or fragile mechanics rather than drawbacks of the sensing principle.

#### 4.1.4. Spectroscopy (Table 3)

The scores clustered around mid-range, despite a strong detection potential in specific works. Top entries, such as Early aphid detection (5,4,3), showed that the performance and capital cost can be competitive, while the implementability is sensitive to the calibration stability, illumination control, and thermal conditions in outdoor scenarios. Studies that improved the portability tended to moderate *P* or increase the acquisition complexity. The overall signal aligned with the strengths in early or non-visible cues under controlled or semi-controlled acquisition.

#### 4.1.5. Distributional Analysis of PCI Across Domains

Table 5 reports AI at a median of 3.20 with an IQR of [2.60,3.60], sensors at 3.17 with an IQR of [2.75,3.67], and spectroscopy at 2.60 with an IQR of [2.40,3.10]. The boxplots in Figure 5 show overlapping distributions for AI and sensors, which is consistent with their close medians and similar dispersion. Spectroscopy trailed primarily through lower implementability, and exhibited a narrower spread that concentrated values below the upper quartiles of AI and sensors. The proportion above a practical readiness threshold of PCI≥3.5 was 32.0% for AI and 33.33% for sensors, compared with 18.18% for spectroscopy (Table 5). This contrast supports the interpretation that AI and well-engineered sensors more often reach deployment-leaning profiles under the chosen rubric.

#### 4.1.6. Pareto View of Trade-Offs

Reading directly from Table 1, Table 2 and Table 3, compact non-dominated sets summarize the feasible design choices within each domain. For sensors, (4,4,4) and (2,5,4) capture balanced versus ultra-lean philosophies. For AI, clusters near (4,5,4) group detector and attention models that combine the task performance with deployability. For spectroscopy, (5,4,3) and (4,3,4) reflect an early-detection versus portability tension. These contrasts describe how *P*, *C*, and *I* interact under the stated weights and explain the observed distributional ordering across domains.

### 4.2. Inter-Rater Agreement

#### 4.2.1. Inter-Rater Agreement (PA vs. Author 1): Interpretation

The agreement profile in Table 6 exhibited a coherent and domain-consistent pattern. First, quadratically weighted κ exceeded the linear variant across all cells, indicating that most disagreements were adjacent on the five-level ordinal scale (i.e., near misses rather than divergent judgements). This behaviour is exactly what one expects when raters share a common rubric, but vary slightly in anchoring.

Sensors. The agreement was substantial for the performance ($\kappa_{\mathrm{quad}}=0.733$) and implementability (0.800), while the cost was somewhat lower (0.621). This pattern is consistent with a heterogeneous evidence base: deployments, platforms, and environmental conditions differ widely, which can blur methodological clarity even when the intended purpose and the practical impact are described in comparable terms. In other words, raters tend to concur on what the work aims to achieve and why it matters, but fine-grained protocol clarity shows more room for interpretation.

Spectroscopy. The agreement was very high for the cost ($\kappa_{\mathrm{quad}}=0.925$) and implementability (0.849), and lower for the performance (0.569). This was also aligned with the expectations: spectroscopy papers often report instrument settings, acquisition ranges, and pre-processing steps in a structured way, favouring convergent judgements on clarity and downstream impact, while the framing of purpose can span broader application spaces (e.g., screening vs. diagnosis vs. quantification), leaving more latitude to interpretation.

Pooled across domains. The combined estimates (sensors+spectroscopy) remain substantial for all dimensions ($\kappa_{\mathrm{quad}}=0.726$–0.827), reinforcing that the agreement signal is stable and not dominated by a single domain. Taken together, these trends indicate consistent rating behaviour: a high concordance where information is concretely specified (clarity/impact in spectroscopy; purpose/impact in sensors) and smaller, adjacent disagreements where conceptual framing invites nuance.

Two caveats qualify this interpretation. First, the sample sizes per domain were modest (n=11–18), so the confidence intervals would have been relatively wide; nonetheless, the same adjacency pattern (quadratic > linear) appears to be uniform. Second, we preserved the full five-level grid even when some categories were infrequently used, which can slightly depress the linear κ, yet leave the substantive conclusion unchanged (adjacent disagreements dominate). Practically, the weakest cells (performance in spectroscopy; cost in sensors) mark clear targets for a brief post hoc calibration without undermining the overall reliability.

#### 4.2.2. Inter-Rater Agreement (PA vs. Author 2): Interpretation

The agreement profile in Table 7 (imaging/AI only; n=25 per dimension) shows modest concordance overall, with a clear adjacency signal in two of the three dimensions. Specifically, quadratically weighted κ exceeded the linear variant for cost ($\kappa_{\mathrm{quad}}\;=\;0.320$ vs. $\kappa_{\mathrm{lin}}\;=\;0.144$) and implementability (0.444 vs. 0.310), indicating that most disagreements in these dimensions were adjacent on the five-level ordinal scale (near-miss ratings). For performance, $\kappa_{\mathrm{quad}}$ was slightly below $\kappa_{\mathrm{lin}}$ (0.299 vs. 0.314), suggesting a small number of non-adjacent discrepancies (e.g., two-step differences).

Among the three dimensions, implementability attained the highest concordance ($\kappa_{\mathrm{quad}}\;=\;0.444$), consistent with a shared reading of deployment cues (e.g., evidence beyond lab-only datasets, on-device inference class, environmental realism). Cost showed a fair agreement (0.320), likely reflecting occasional ambiguity in how papers specify inference hardware or map it to the rubric’s cost anchors. Performance had the lowest $\kappa_{\mathrm{quad}}$ (0.299), which we attribute to heterogeneity in the reported metrics (mAP vs. F1/accuracy), task framing (detection vs. classification), and threshold choices; these factors can induce sporadic two-step scoring gaps.

The main caveat of this interpretation is the fact that we preserved the full five-level grid even when certain score levels were rarely used, which can depress the linear κ while leaving the qualitative takeaway intact (adjacent disagreements dominate in C and I; occasional non-adjacent differences in P).

### 4.3. Decision Maps: From PCI to Field Choices

We translated the PCI evidence into a set of deployment cases that function as operational playbooks. Each case begins with a threshold tuple $\left(t_{P},t_{C},t_{I}\right)$ used as an illustrative, non-prescriptive example to capture typical trade-offs; it is a pragmatic (i.e., arbitrary for this demonstration) choice rather than a fixed standard. The case then routes through binary prompts to a sensing modality and to cited exemplars. These thresholds simply make explicit how the cost, performance, and implementability interact under common constraints, and should be re-parameterized to match the reader’s own power, budget, and O&M realities.

Candidate papers within each map were drawn from Table 1, Table 2 and Table 3 and satisfied the case-specific $\left(t_{P},t_{C},t_{I}\right)$. Selection was representative rather than exhaustive to keep the guidance actionable, and every recommendation is traceable to the PCI tables. Each subsubsection presents one case with a short rationale and the corresponding figure.

#### 4.3.1. Case 1—Low-Power, Rapid Implementability

**Figure 6:**
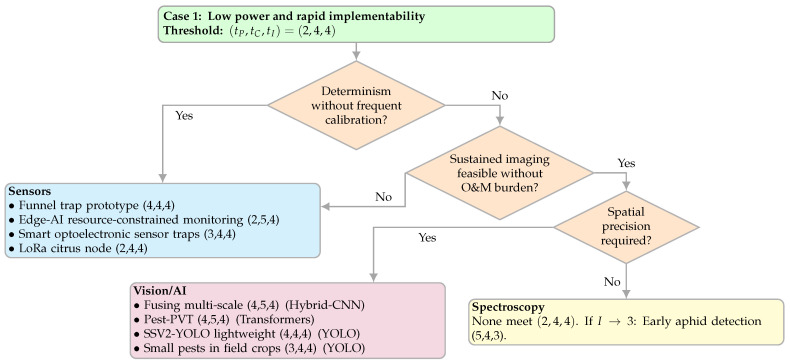
Decision map for Case 1 (low power and rapid implementability). Threshold uses $\left(t_{P},t_{C},t_{I}\right)=\left(2,4,4\right)$ [20,26,35,37,45,47,48,52,63].

This case covers low-power deployments (e.g., small solar), long unattended intervals, and intermittent connectivity, where sensing is event-driven or periodic rather than continuous high-throughput. Typical examples are fixed nodes expected to run for weeks with minimal field visits. We pre-filtered the candidates with the tuple $\left(t_{P},t_{C},t_{I}\right)=\left(2,4,4\right)$ on the PCI rubric (Section 2.4.1); this means an adequate task performance, a high cost-efficiency at the inference/hardware, and a high implementability under field conditions. The tuple is a filter, not a ranking step. Figure 6 shows the decision map.

Figure 6 routes among modality families using a short sequence of binary prompts aligned with this case. Here, the O&M burden means the routine work to keep a node within spec (e.g., cleaning optics, desiccants, restarts, enclosure checks, OTA), with visit cadence as a practical cue. Determinism denotes the measurement stability without frequent recalibration (low day–night drift, no strict reflectance references or long warm-up, predictable SNR under dust/wind/noise). Sustained imaging is the ability to maintain camera or UAV acquisition given available Wh/day and staff effort; if this is not feasible at a low O&M, non-imaging sensors are generally preferred under (2,4,4). The spatial precision is the required action granularity, from instance/leaf-level localisation (counts, boxes) to plot/node-level indicators. Note that the cost component differs by modality (in AI, it reflects the inference hardware class; in sensors/spectroscopy, it reflects the bill of materials/instrumentation). The “sensors” leaf includes opto/electronic traps with onboard vision when engineered as self-contained nodes. The spectroscopy panel is shown for context: under (2,4,4), it is typically filtered out by implementability and becomes relevant only if that requirement is relaxed (e.g., $t_{I}\to 3$).

#### 4.3.2. Case 2—High-Accuracy, Cost-Flexible

**Figure 7:**
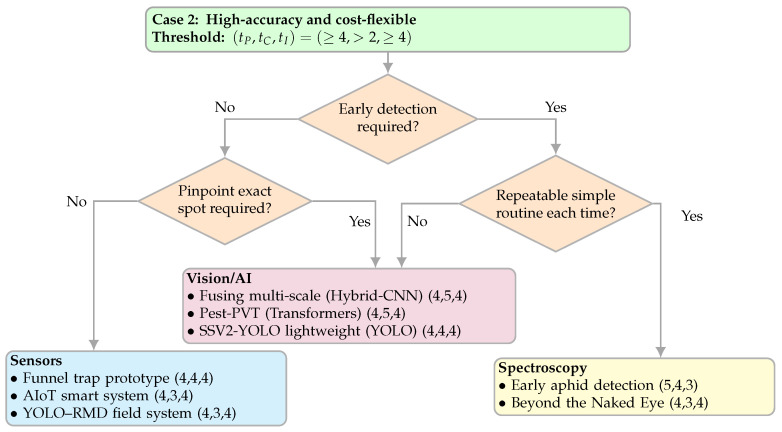
Decision map for Case 2 (high accuracy and cost-flexible). Threshold uses $(t_{P},t_{C},t_{I})=$(≥4,>2,≥4). [20,26,35,45,46,49,63,64].

This case targets settings where a very high detection accuracy is required and implementability under field conditions must remain strong, while the cost is not the primary constraint (e.g., high-value crops, zero-tolerance thresholds, greenhouse or export-oriented operations). Typical examples include programs that demand reliable calls with minimal ambiguity, under periodic or event-driven scouting. We pre-filtered candidates with the tuple $\left(t_{P},t_{C},t_{I}\right)=\left(\geq 4,>2,\geq 4\right)$ on the PCI rubric (Section 2.4.1); this means a high task performance and high implementability, with a cost requirement above minimal. The tuple is a filter, not a ranking step. Figure 7 shows the decision map.

Figure 7 guides the choice through simple binary prompts. “Early detection” means detecting before damage is visible. “Repeat a simple routine” means measuring in a similar way each time (roughly the same place/time/distance or a consistent sampling routine) without complicating field work; if that is feasible, spectroscopy is preferred when early detection matters. “Pinpoint the exact spot” means the system must indicate exactly where to act (plant/leaf/insect), in which case vision/AI is preferred. When early detection is not required and pinpointing is not needed, sensors can be appropriate as plot-/node-level indicators.

#### 4.3.3. Case 3—Block-Scale Routine Scouting

**Figure 8:**
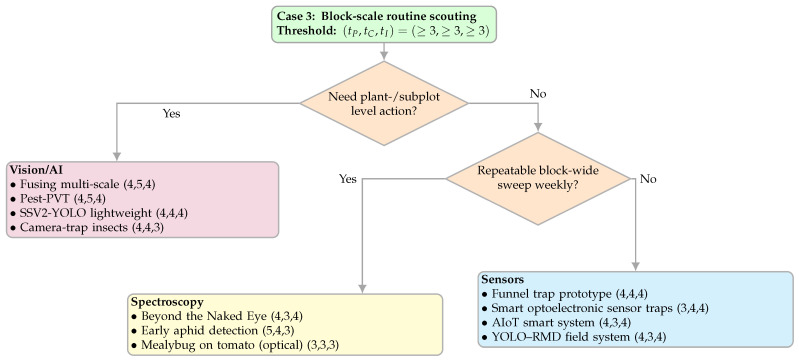
Decision map for Case 3 (block-scale routine scouting). Threshold uses $\left(t_{P},t_{C},t_{I}\right)$=(≥3,≥3,≥3). Each path routes to a single modality; within each leaf, choose by $PCI_{\mathrm{final}}$ (Section 3). [20,26,35,36,45,46,48,49,63,64,66].

This case targets routine scouting across large blocks with light, periodic visits and mid-tier budgets/power. The emphasis is on crew capacity and coverage at the block scale rather than instrument internals. We pre-filtered candidates with the tuple $\left(t_{P},t_{C},t_{I}\right)=\left(\geq \;3,\geq \;3,\geq \;3\right)$ on the PCI rubric (Section 2.4.1). Figure 8 shows the decision map.

Figure 8 routes using two prompts tuned to personnel and scale. “Need plant-/subplot level action?” determines whether interventions require precise localisation at the plant or subplot granularity (actionable coordinates) versus area-level cues. “Repeatable block-wide sweep weekly?” captures whether the crew can execute a simple, low-maintenance pass at weekly cadence. Paths lead to a single modality per exit: Vision/AI is used for fine-grained action; spectroscopy is used when a stable block-wide routine is feasible without heavy maintenance; and sensors are used when coverage is partial or routines are irregular and persistent stations are preferable.

#### 4.3.4. Operational Guidance and Minimum Reporting Set

The PCI lens and decision maps in this review are meant to support field choices rather than rank algorithms in the abstract. The maps are illustrative templates, not prescriptions: the thresholds $\left(t_{P},t_{C},t_{I}\right)$, prompts, and exemplars shown are examples that readers should re-parameterize to their own budget/power/maintenance constraints, edit (adding/removing branches), or use to synthesize new maps for their specific use case. The papers listed at each leaf are representative, not exhaustive; inclusion does not imply endorsement, and omission is not a negative judgment. We recommend a case-by-case appraisal of candidates (crop–pest context, scale, climate, acquisition workflow, communications, power, O&M cadence, regulatory factors), using the minimum PCI reporting set in Section 4.3.4, and, where feasible, small pilots to validate the performance under local conditions. Practitioners should (i) set a constraint tuple $\left(t_{P},t_{C},t_{I}\right)$, (ii) follow and/or adapt the relevant map (Section 4.3), (iii) resolve ties at a leaf using $PCI_{\mathrm{final}}$ (Section 3) and the lowest O&M burden compatible with the required action granularity (leaf/plant/subplot/block), and (iv) document any adaptations so their choices remain reproducible.

Scenario selection (Cases 1–3).

- Case 1 with $\left(t_{P},t_{C},t_{I}\right)=\left(2,4,4\right)$: low power, infrequent visits, intermittent connectivity. Route by measurement determinism, feasibility of sustained imaging without added O&M burden, and required spatial precision. Default to non-imaging sensors if sustained imaging at low O&M is not feasible; choose lightweight vision/AI when pinpoint action is required.
- Case 2 with $\left(t_{P},t_{C},t_{I}\right)=\left(\geq \;4,>\;2,\geq \;4\right)$: very high accuracy is critical and implementability must remain strong while cost is flexible. Prefer spectroscopy when early (pre-symptomatic) detection is needed under a simple, repeatable routine; prefer vision/AI when exact intervention points are required; otherwise, sensors can serve as plot-/node-level indicators.
- Case 3 with $\left(t_{P},t_{C},t_{I}\right)=\left(\geq \;3,\geq \;3,\geq \;3\right)$: block-scale routine scouting under mid-tier budgets and power, emphasizing coverage and crew capacity over instrument internals.

Tie-breaks: if early detection is mandatory, choose Case 2; if power/visit constraints are severe, choose Case 1; otherwise, use Case 3. In ambiguous settings, start from the closest case and re-parameterize $\left(t_{P},t_{C},t_{I}\right)$ to local constraints.

Minimum reporting set (to enable reproducibility and PCI scoring).

- Power/compute envelope: inference hardware class, typical Wh/day, duty cycling.
- Acquisition and calibration: illumination control, references/warm-up, per-visit routine.
- Environmental robustness: enclosure/IP, optics maintenance, condensation/dust handling.
- Backhaul and fleet ops: link type, duty cycle, time sync/OTA, remote resets.
- O&M cadence: visit frequency, minutes/visit, consumables/spares.
- Action granularity: detection unit and mapping to interventions.
- Evidence setting: training vs. deployment domains, occlusion/background variability, seasonality.
- Cost ranges: BOM class and any inference-time computing cost assumptions.

This compact checklist is intended to accompany future studies across modalities so that results are directly comparable under deployment constraints, not only in accuracy terms.

### 4.4. Limitations

While this systematic review aimed for methodological rigor and technical depth, several limitations should be acknowledged:

- Database Coverage: The search was conducted using IEEE Xplore and Scopus, which collectively provide extensive access to high-impact literature in engineering, computer science, and applied agricultural technologies, including many ACM and Springer publications. Nevertheless, some niche or regional outlets not indexed in these databases may have been excluded.
- Exclusion of Gray Literature: This review deliberately excluded non-peer-reviewed sources such as theses, patents, and technical reports. While this decision supports methodological consistency, it may overlook practical implementations and emerging innovations not yet published in academic venues.
- Scope Restriction to Edible Crops: Studies involving ornamental, industrial, or non-edible crops were excluded to maintain a focused analysis on solutions relevant to food production systems. While justified by the objective, this may have omitted transferable insights from other agricultural domains.
- Assumption of Reported Metrics: Performance indicators (e.g., accuracy, inference time) were accepted as reported by the original authors. Due to the nature of the review, it was not feasible to independently verify these results or the underlying methodologies. However, the evaluation framework penalizes weak or insufficiently documented approaches.
- Subjectivity in Scoring Model: The PCI model introduced in this work is inherently subjective, particularly in its weighting of the performance, cost, and implementability. While these weights are clearly defined and the results are presented both pre- and post-weighting, the model reflects the author’s perspective on trade-offs relevant to practical deployment.
- Implementation Criteria: Inclusion required at least some level of implementation or evaluation (e.g., hardware deployment, simulation with real datasets). Purely theoretical or conceptual works without a clear path for deployment were excluded, which may have left out valuable exploratory contributions.
- AI sub-sampling bias: Within AI, we restricted comparisons to the five most-cited papers per subcategory; this pragmatic filter can favour older/high-visibility venues and may have under-represented recent, yet deployable, designs.
- No formal risk-of-bias tool for AI: We relied on reported metrics and implementation metadata; a domain-specific risk-of-bias instrument for ML in agriculture would improve the comparability across studies.
- Generality of the maps: The decision maps were derived from the current corpus and weights; they were intended as starting points, not prescriptive rules, and should be stress-tested in prospective field deployments across crops and seasons.

## 5. Conclusions

This review reframes pest detection for edible crops around a modality-aware performance–cost–implementability (PCI) lens. Rather than asking which single algorithm or instrument is “best”, we evaluated whether vision/AI, spectroscopy, or indirect sensor systems are fit for purpose under real deployment constraints (power/compute envelopes, acquisition and calibration routines, enclosure/robustness, backhaul and fleet operations, and O&M cadence). Using category-specific PCI weights (Section 2.4.1), we synthesized 2015–2025 evidence into compact decision maps (Section 4.3) intended for implementation choices in the field.

Three consistent signals emerged. First, under our weighting policy, imaging (AI) and well-engineered sensor systems more frequently achieved deployment-leaning profiles, with a median (IQR) PCI of 3.20 [2.60–3.60] and 3.17 [2.75–3.67], respectively, compared with 2.60 [2.40–3.10] for spectroscopy (Table 5, Figure 5). Roughly one-third of the AI (32.0%) and sensor works (33.33%) exceeded a practical-readiness threshold (PCI≥3.5), versus 18.18% for spectroscopy. Second, the Pareto views clarified the trade-offs: detectors and attention-based models near (P,C,I)≈(4,5,4), sensor nodes spanning balanced (4,4,4) or ultra-lean (2,5,4) philosophies, and spectroscopy split between early-detection strength (5,4,3) and portability (4,3,4). Third, when translated into decisions, low-power, rapid-deployment scenarios (Case 1) typically favoured sensors or lightweight detectors; high-accuracy, cost-flexible programs (Case 2) favoured spectroscopy for pre-symptomatic cues if a simple, repeatable routine was feasible, or vision/AI when pinpointing is required; and block-scale routine scouting (Case 3) routed to a single modality per operational prompt.

The inter-reviewer reliability supported the robustness of these synthesis steps. For the Primary Author (PA) vs. Author 1, the quadratically weighted Cohen’s κ was substantial to very high across domains and dimensions (Table 6). Most disagreements were adjacent on the five-point scales, which is the pattern expected when a shared rubric is applied consistently. For the PA vs. Author 2, the agreement was modest and limited to imaging/AI (Table 7; n=25), with $\kappa_{\mathrm{quadratic}}\approx 0.30$–0.44. Disagreements were primarily adjacent in cost and implementability, whereas performance showed occasional non-adjacent differences. This profile indicates a coherent rubric with room for minor calibration (for example, metric anchoring for performance and mapping hardware classes for cost). Because the PA vs. Author 2 covers only one domain, these estimates are not directly comparable to the PA vs. Author 1.

Practically, we recommend that future studies across modalities report a minimum PCI metadata set: (i) inference hardware class and Wh/day; (ii) acquisition and calibration routine (illumination control, references, warm-up); (iii) enclosure/IP and optics maintenance; (iv) backhaul, duty-cycling, synchronization, and OTA; (v) O&M cadence and minutes/visit; (vi) action granularity (leaf/plant/subplot/block); (vii) evidence setting and domain shift; and (viii) BOM/cost class. Adopting this minimum set will make cross-study comparisons reproducible and deployment-oriented, rather than accuracy-only. Together, the weighted PCI scores, decision maps, and reporting checklist offer a deployment-oriented approach that supports modality selection under real-world constraints, enabling practitioners to identify acceptable systems without relying solely on accuracy.

This work has limitations. The PCI weights reflect our deployment-centric stance; spectroscopy’s mid-range PCI does not negate its value for early, non-visible cues, but highlights integration frictions under field variability. Our AI sub-sampling by citations is pragmatic and may have under-represented recent deployable designs. The reported metrics were accepted at face value. Nonetheless, the convergence of distributional results, Pareto structure, decision maps, and substantial inter-rater agreement gives confidence in the main conclusions.

Looking ahead, we see four priorities: (i) A community risk-of-bias instrument tailored to ML and sensing for agriculture. (ii) Prospective, multi-season field trials that expand the evidence base for tuple selections and refine the binary questions in our decision maps. In our framework, most terms reflect implementability and real-world constraints such as power, labour, and connectivity, but temporal factors (e.g., seasonal variability, calibration drift, and the annotation burden across crop cycles) are not yet systematically captured. Multi-season trials would therefore provide the missing longitudinal evidence needed to translate PCI scoring into deployment guidance. (iii) Uncertainty-aware, on-device inference for actionable thresholds. (iv) Living PCI templates and decision maps that update as modalities and embedded platforms evolve. For practitioners, the takeaway is straightforward: select the sensing modality that matches your power, labour, connectivity, and action-granularity constraints—PCI and the provided maps translate published evidence into field-ready choices, accelerating the time to deployment beyond accuracy-only benchmarks.

## Figures and Tables

**Table 1:** Raw PCI component scores (P,C,I) and the resulting weighted ${PCI}_{\mathrm{final}}$ for pest-detection articles in the imaging (AI) category.

Article	P	C	I	${\mathrm{PCI}}_{\mathrm{final}}$
CNN				
An Entire-and-Partial Feature Transfer Learning Approach for Detecting the Frequency of Pest Occurrence [21]	4	3	3	3.6
Multi-task learning model for agricultural pest detection from crop-plant imagery: A Bayesian approach [22]	3	3	2	2.75
PestNet: An End-to-End Deep Learning Approach for Large-Scale Multi-Class Pest Detection and Classification [23]	3	2	2	2.6
Insect detection and classification based on an improved convolutional neural network [24]	3	2	2	2.6
Transfer Learning-Based Framework for Classification of Pest in Tomato Plants [25]	3	2	2	2.6
Hybrid CNN				
Fusing multi-scale context-aware information representation for automatic in-field pest detection and recognition [26]	4	5	4	4.15
Multiple Diseases and Pests Detection Based on Federated Learning and Improved Faster R-CNN [27]	4	3	3	3.6
Multi-model LSTM-based convolutional neural networks for detection of apple diseases and pests [28]	4	2	2	3.2
Research on Recognition Model of Crop Diseases and Insect Pests Based on Deep Learning in Harsh Environments [29]	3	2	3	2.85
MSR-RCNN: A Multi-Class Crop Pest Detection Network Based on a Multi-Scale Super-Resolution Feature Enhancement Module [30]	3	2	2	2.6
Transformers				
Pest-PVT: A model for multi-class and dense pest detection and counting in field-scale environments [20]	4	5	4	4.15
A New Pest Detection Method Based on Improved YOLOv5m [31]	4	4	3	3.75
TP-YOLO: A Lightweight Attention-Based Architecture for Tiny Pest Detection [32]	4	3	3	3.6
A Study on Tomato Disease and Pest Detection Method [33]	4	3	2	3.35
HCFormer: A Lightweight Pest Detection Model Combining CNN and ViT [34]	4	3	2	3.35
YOLO				
A lightweight SSV2-YOLO based model for detection of sugarcane aphids in unstructured natural environments [35]	4	4	4	4
Accurate detection and identification of insects from camera trap images with deep learning [36]	4	4	3	3.75
Small Pests Detection in Field Crops Using Deep Learning Object Detection [37]	3	4	4	3.4
Deep Learning Based Detector YOLOv5 for Identifying Insect Pests [38]	4	2	2	3.2
Maize-YOLO: A New High-Precision and Real-Time Method for Maize Pest Detection [39]	3	3	2	2.75
Others				
Detection of whitefly pests in crops employing image enhancement and machine learning [40]	4	3	2	3.35
AF-RCNN: An anchor-free convolutional neural network for multi-categories agricultural pest detection [41]	3	2	2	2.6
Crop pest recognition based on a modified capsule network [42]	3	2	2	2.6
Plant Diseases and Pests Detection Using Machine Learning [43]	2	2	2	2.0
A Stable Diffusion Based Image Generation Method for Few-Shot Emerging Pest Detection in the Wild [44]	2	1	1	1.6

**Table 2:** Raw PCI component scores (P,C,I) and the resulting weighted $PCI_{\mathrm{final}}$ for pest-detection articles in the sensors category.

Article	P	C	I	${\mathrm{PCI}}_{\mathrm{final}}$
Automatic Detection of Moths (Lepidoptera) with a Funnel Trap Prototype [45]	4	4	4	4.00
An AIoT Based Smart Agricultural System for Pests Detection [46]	4	3	4	3.67
A Novel Resource-Constrained Insect Monitoring System based on Machine Vision with Edge AI [47]	2	5	4	3.67
Automated Surveillance of Lepidopteran Pests with Smart Optoelectronic Sensor Traps [48]	3	4	4	3.67
An Intelligent Field Monitoring System Based on Enhanced YOLO-RMD Architecture for Real-Time Rice Pest Detection and Management [49]	4	3	4	3.67
Handheld Crop Pest Sensor Using Binary Catalyst-Loaded Nano-SnO_2_ Particles for Oxidative Signal Amplification [50]	4	4	3	3.67
A high performance-oriented AI-enabled IoT-based pest detection system using sound analytics in large agricultural field [51]	4	4	2	3.33
Integrating IoT and Image Processing for Crop Monitoring: A LoRa-Based Solution for Citrus Pest Detection [52]	2	4	4	3.33
Unified Pest Prevention and Control System based on AIoT for Sustainable Agriculture [53]	3	3	4	3.33
A self-built electronic nose system for monitoring damage caused by different rice planthopper species [54]	4	3	2	3.00
IoT Meets Computer Vision: An Improved Detection of Tomato Pests and Diseases [55]	4	3	2	3.00
Prototyping a Low-Cost Pest Detection System for CNT0718-26-1-1-1 Rice Farming [56]	2	4	3	3.00
Transfer Learning and Object Detection for Improved Date Fruit Pest Recognition [57]	4	3	2	3.00
Shadow Effect for Small Insect Detection by W-Band Pulsed Radar [58]	2	4	2	2.67
CHILI PLANT MONITORING SYSTEM USING YOLO OBJECT DETECTION TECHNOLOGY [59]	2	4	2	2.67
Twin-Layer Deep Learning Model with IoT Integration for Intelligent Pest Detection in Smart Farming [60]	2	3	2	2.33
Towards noise robust acoustic insect detection: from the lab to the greenhouse [61]	2	2	2	2.00
TL-AGRO: Transfer Learning-based Perilous Insect Detection Framework for Smart Agriculture Underlying Edge Computing [62]	2	3	1	2.00

**Table 3:** Raw PCI component scores (P,C,I) and the resulting weighted $PCI_{\mathrm{final}}$ for pest-detection articles in the spectroscopy category.

Article	P	C	I	${\mathrm{PCI}}_{\mathrm{final}}$
Early pest Detection of Aphid Infestation [63]	5	4	3	4.00
Beyond the Naked Eye: Computer Vision for Detecting Brown Marmorated Stink Bug and Its Punctures [64]	4	3	4	3.80
Hyperspectral imaging combined with convolutional neural network for outdoor detection of potato diseases [65]	4	2	3	3.20
Detecting early mealybug infestation stages on tomato plants using optical spectroscopy [66]	3	3	3	3.00
A novel method for detection of Pieris rapae larvae on cabbage leaves using NIR hyperspectral imaging [67]	4	2	2	2.80
Differentiation of Wheat Diseases and Pests Based on Hyperspectral Imaging Technology with a Few Specific Bands [68]	3	3	2	2.60
Nondestructive Detection of Litchi Stem Borers Using Multi-Sensor Data Fusion [69]	4	1	2	2.60
Pest Detection and Classification in Peanut Crops Using CNN, MFO, and EViTA Algorithms [70]	3	2	2	2.40
Hyperspectral Characterization of Coffee Leaf Miner (Leucoptera coffeella) (Lepidoptera: Lyonetiidae) Infestation Levels: A Detailed Analysis [71]	3	2	2	2.40
Detection of Leaf Miner Infestation in Chickpea Plants Using Hyperspectral Imaging in Morocco [72]	3	2	2	2.40
Detection of spotted wing drosophila hidden infestation in blueberry using hyperspectral imaging and machine learning [73]	3	2	2	2.40

**Table 4:** Imaging (AI) subcategories ranked by median PCI under the category-specific weights (AI: 0.60/0.15/0.25). Values are median $\left[Q_{1}–Q_{3}\right]$, IQR $=Q_{3}\;-\;Q_{1}$, and mean ± SD.

Subcategory	*n*	Median [$Q_{1}$–$Q_{3}$]	IQR	Mean ± SD
Transformers	5	3.60 [3.35–3.75]	0.40	3.64 ± 0.33
YOLO	5	3.40 [3.20–3.75]	0.55	3.42 ± 0.49
H-CNN	5	3.20 [2.85–3.60]	0.75	3.28 ± 0.62
CNN	5	2.60 [2.60–2.75]	0.15	2.83 ± 0.44
Others	5	2.60 [2.00–2.60]	0.60	2.43 ± 0.67

**Table 5:** Aggregate PCI statistics by domain under the category-specific weighting policy.

Domain	*n*	Median [IQR]	Mean ± SD	PCI≥3.5 (%)
AI	25	3.20 [2.60–3.60]	3.12 ± 0.65	32.0
Sensors	18	3.17 [2.75–3.67]	3.11 ± 0.59	33.33
Spectroscopy	11	2.60 [2.40–3.10]	2.87 ± 0.57	18.18

**Table 6:** Linearly and quadratically weighted Cohen’s κ for inter-rater agreement (PA vs. Author 1), computed per modality domain (sensors, spectroscopy, combined) and PCI dimension (performance, cost, implementability). Each domain spans three rows, one per PCI axis. Columns indicate $\kappa_{\mathrm{linear}}$ and $\kappa_{\mathrm{quadratic}}$ agreement on a five-level ordinal scale. Sample sizes: sensors n=18, spectroscopy n=11, combined n=29.

Domain	Metric	$\kappa_{\mathrm{linear}}$	$\kappa_{\mathrm{quadratic}}$
Sensors	P	0.658	0.733
	C	0.503	0.621
	I	0.638	0.800
Spectroscopy	P	0.463	0.569
	C	0.889	0.925
	I	0.776	0.849
Sensors + Spectroscopy	P	0.632	0.726
	C	0.665	0.764
	I	0.695	0.827

**Table 7:** Linearly and quadratically weighted Cohen’s κ for inter-rater agreement (PA vs. Author 2), computed for the imaging (AI) domain across the three PCI dimensions (performance, cost, implementability). Rows list the PCI axes, and columns report $\kappa_{\mathrm{linear}}$ and $\kappa_{\mathrm{quadratic}}$ agreement on a five-level ordinal scale. Sample size: imaging (AI) n=25.

Domain	Metric	$\kappa_{\mathrm{linear}}$	$\kappa_{\mathrm{quadratic}}$
Imaging (AI)	P	0.314214	0.299065
	C	0.143836	0.319728
	I	0.310345	0.444444

## Data Availability

No new data were created or analyzed in this study.

## Abbreviations

The following abbreviations are used in this manuscript:

PCI	Performance–cost–implementability
CNN	Convolutional neural network
AI	Artificial intelligence
